# Protocol for economic evaluation alongside the IMPLEMENT cluster randomised controlled trial

**DOI:** 10.1186/1748-5908-3-12

**Published:** 2008-02-22

**Authors:** Duncan Mortimer, Simon D French, Joanne E McKenzie, Denise A O'Connor, Sally E Green, the IMPLEMENT study group

**Affiliations:** 1 Centre for Health Economics, Faculty of Business & Economics, Monash University, Melbourne, Australia; 2 Health Economics, Division of Health Sciences, University of South Australia, Adelaide, Australia; 3 Monash Institute of Health Services Research, Monash University, Melbourne, Australia

## Abstract

**Background:**

The recent development and publication of evidence-based clinical practice guidelines (CPGs) for acute low back pain (LBP) has resulted in evidence-based recommendations that, if implemented, have the potential to improve the quality and safety of care for acute LBP. While a strategy has been specified for dissemination of the CPG for acute LBP in Australia, there is no accompanying plan for active implementation. Evidence regarding the cost-effectiveness of active implementation of CPGs for acute LBP is sparse. The IMPLEMENT study will consider the incremental benefits and costs of progressing beyond development and dissemination to implementation.

**Methods/design:**

Cost-effectiveness and cost-utility analyses alongside the IMPLEMENT cluster randomised controlled trial (CRCT) from a societal perspective to quantify the additional costs (savings) and health gains associated with a targeted implementation strategy as compared with access to the CPG via dissemination only.

**Discussion:**

The protocol provided here registers our intent to conduct an economic evaluation alongside the IMPLEMENT study, facilitates peer-review of proposed methods and provides a transparent statement of planned analyses.

**Trial registration:**

Australian New Zealand Clinical Trials Registry ACTRN012606000098538

## Background

### The IMPLEMENT Study

The recent development and publication of evidence-based clinical practice guidelines (CPGs) for acute low back pain (LBP) has resulted in evidence-based recommendations that, if implemented, have the potential to improve the quality and safety of care for acute LBP [1]. While a strategy has been specified for dissemination of the CPG for acute LBP in Australia, there is as yet no accompanying plan for active implementation. The IMPLEMENT study will consider the incremental benefits and costs of progressing beyond development and dissemination to implementation, and has the following objectives: to develop a targeted strategy for implementing CPG for acute LBP into Australian general practice; to test the effectiveness of the strategy for implementing the CPG for acute LBP, with respect to both general practitioners' (GPs) practice and patient outcomes, by conducting a cluster randomised controlled trial (CRCT); and, to determine the cost-effectiveness of the developed strategy as compared to current practice.

The purpose of the present paper is to describe methods for the cost-effectiveness analysis alongside the CRCT. Detailed descriptions of methods for development of the targeted implementation strategy and design of the CRCT in the IMPLEMENT study are given elsewhere [2].

### Economics of implementation

It is now well established that development and dissemination of CPGs will not necessarily produce improvements in clinical practice [3]. Clinical practice has proved resilient to recommendations for practice change embedded in a CPG even where the gap between current and best practice care equates to a clinically significant difference in patient outcomes [3]. Where development and dissemination of a CPG requires substantial investment and where further expenditure on implementation might be required to effect any change in practice [4], there is a clear imperative to understand the cost-effectiveness of competing strategies for practice change.

Despite this imperative, a recent review of 63 economic evaluations and cost analyses conducted alongside rigorous experimental studies of guideline implementation strategies published between 1966 and 1998 concluded that the available economic evidence was lacking in methodological rigour and often drew conclusions that 'must clearly be treated with suspicion' [4]. A significant number of cost-effectiveness analyses of guideline implementation strategies have been published in the subsequent period from 1998 to present [5-9]. Of these, just one study directly considered the effectiveness and efficiency of strategies for the implementation of a CPG for non-specific LBP. Hoeijenbos *et al*. [9] conducted a cost-utility analysis alongside a CRCT comparing active implementation plus standard dissemination of the Royal Dutch Physiotherapy Association guideline for non-specific LBP against standard dissemination alone in a sample of 113 physiotherapists and 483 patients drawn from 68 practices [10]. The active implementation strategy in the Dutch trial consisted of an initial training session with presentation and discussion of the guideline plus 'special skills' practice, and a follow-up session four weeks later to discuss experience or problems identified while practicing according to the guideline. Standard dissemination consisted of mail distribution of the guideline together with self-evaluation forms to assess whether current management was consistent with the guideline, additional materials including forms designed to facilitate peer-to-peer discussion and a copy of the Quebec Back Pain Disability Scale. Physiotherapists in both arms of the trial may also have been aware of discussion around the development of the guideline in local professional journals.

Hoeijenbos *et al*. [9] calculated the incremental treatment cost of active implementation over standard dissemination at €366 per physiotherapist for roll-out of the active implementation strategy to 50% of the 12,687 physiotherapists working in primary care in the Netherlands. Total healthcare utilisation reported at six week follow-up was significantly lower for patients in the intervention arm (mean = €125, SD = €91) than for patients in the control group (mean = €145, SD = €95, p = 0.026). Given treatment of a sufficient number of patients per physiotherapist, the incremental treatment cost associated with active implementation might well be recovered through savings in healthcare utilisation in the initial six weeks of follow-up. However, the observed savings in healthcare utilisation in the initial six weeks of follow-up could not be attributed to the intervention, because randomisation of physiotherapists per practice failed to ensure against pre-existing between-group differences in patient characteristics. EQ5D health-related quality of life scores (HRQoL) for the intervention group at baseline (mean = 0.6730, SD = 0.2042) were significantly higher than for the control group (mean = 0.6134, SD = 0.2661, p = 0.006), with much of this difference attributable to a difference on the self-care dimension of the EQ5D in favour of the intervention group. Hoeijenbos *et al*. conclude that 'it is very likely that the extended implementation strategy incurs extra costs without producing health gains, hence it is very likely to be not cost-effective' [9]. While controlling for observed differences in quality of life at baseline would be unlikely to alter this conclusion, it is possible that between-group differences at baseline were also present in one or more unobserved patient characteristics.

While the principles of economic evaluation for health care interventions are now well-established, the literature provides relatively few examples of well-executed economic evaluations of implementation strategies and the characteristics of CPGs raise several issues in the estimation and interpretation of cost-effectiveness [11,12]. The first such issue concerns the breadth of the evaluated intervention. Vale *et al*. [4] suggest that strategies for implementation of a CPG should typically be considered as just one component of a broader strategy to promote best-practice care that would also include development and dissemination of the CPG. They further argue that costs and benefits arising from a combined strategy of development plus dissemination plus implementation should be compared against costs and benefits arising from 'no intervention' except where development and dissemination have themselves been demonstrated to be cost-effective compared to current practice, or where exclusion of development and dissemination can be justified on grounds of perspective or relevance. There may, however, be many circumstances where development and dissemination of the CPG have already been undertaken, and where their costs and benefits have already been reflected in current practice. In such circumstances, practice in the absence of development and dissemination may not be observable. Moreover, the costs and effects associated with development and dissemination of the CPG cannot easily be 'undone' and therefore have little bearing on the subsequent policy decision as to implementation.

The costs of guideline development may be substantial and that development and dissemination alone might have relatively little impact on practice patterns [4]. Comparing a combined strategy of development plus dissemination plus implementation versus 'no intervention' would therefore be expected to yield very different results than would a comparison between the combined strategy and development plus dissemination. Each comparison may, however, be of assistance to policy-makers in a particular policy context. Where development and dissemination have already taken place, the incremental costs and effects associated with implementation are likely to be of greatest interest. Where development and dissemination have not yet taken place, the incremental costs and benefits associated with the combined strategy of development plus dissemination plus implementation as compared to 'no intervention' will be of greatest use to policy-makers.

The second issue concerns the potential for repeated use of various components of the evaluated intervention. The information embedded in a CPG and an implementation strategy is 'non-rival' such that a single use does not diminish the quantity or quality of information available for subsequent use. The development of a CPG therefore amounts to a one-time investment that may find a repeated use in other populations, in subsequent cohorts of practitioners, or in other contexts. Likewise, an implementation strategy may find repeated use in other populations/contexts, in subsequent cohorts, or perhaps even for implementation of another CPG in another disease-area. Drummond *et al*. [13] note the importance of identifying capital outlays (such as expenditure associated with development of the implementation strategy) that should be amortised over the lifetime of the asset. The question then arises: what is the lifetime of the implementation strategy? For non-rival assets bearing repeated and wider use over a sufficiently long lifetime, the cost of a single use approaches zero. It may, however, be appropriate to include the entire cost of development if the usefulness of the implementation strategy is restricted to the disease area, institutional context, practitioner group, or patient population under study, or if the usefulness of the implementation strategy has an expiration date contingent upon the current technology or the existing evidence base.

### Protocol for economic evaluation

A number of recent findings suggest that cost-effectiveness studies in the public domain may represent a biased sample of the population of economic evaluations [14,15]. It is possible that this finding reflects a publication bias, whereby journal editors with a preference for clearly 'positive' or 'negative' results are responsible for a lack of 'intermediate' incremental cost-effectiveness ratios (ICERs) in the public domain. However, it seems more likely that sponsors may be suppressing the publication of intermediate or negative results, or that analysts are 'gaming' modelled evaluations and 'mining' trial data to meet a target cost-effectiveness ratio. Bell *et al*. [14] suggest that registering all economic evaluations prior to their commencement may provide one means of limiting publication bias and/or the suppression of unfavourable results. However, closer scrutiny of methods employed by analysts would be required to limit gaming/mining to meet a target cost-effectiveness ratio. In the past, close scrutiny has been frustrated by a failure to publish a sufficiently detailed description of methods and analyses to permit critical appraisal.

Published trial protocols typically include a section headed 'economic analyses' when such analyses are planned, but the description of methods is often insufficiently detailed to achieve the purpose of a protocol. Without detail, it is not possible to distinguish planned analyses from post-hoc 'mining' or 'gaming' and analysts can freely substitute between outcomes (*e.g*., lower the 'response' required to be classified as a 'responder'), extend the time-horizon (*e.g*., from trial end to full life expectancy) and/or revise the unit cost attached to particular categories of resource use (*e.g*., value volunteer time at the marginal overtime wage rate instead of at zero) [13]. Each of these variations might produce a substantial improvement in the base-case ICER, but it would not be possible to distinguish such variations from planned analyses based on the scant detail contained in many protocols. Publication and peer-review of detailed study protocols for modelled and trial-based cost-effectiveness analyses are therefore proposed to overcome publication bias, permit closer scrutiny of methods, and to provide a strong disincentive for post-hoc 'gaming' and 'mining'.

The impact of moves to improve transparency and rigour in the conduct of modelled and trial-based evaluations will vary depending on the extent of discretion exercised by sponsors over research methods and dissemination of findings, the complexity of the disease-process, and the extent to which evidence gaps necessitate a reliance on assumption and/or lower level evidence [14]. For implementation science, where few studies have included economic analyses and uncertainty surrounds many key parameters [16], the publication and peer-review of detailed protocols for economic analyses might be expected to yield a comparatively greater improvement in transparency and rigour than for disease areas and interventions with an already well-developed evidence base. The methods summarised below provide a protocol for cost-effectiveness analysis alongside the IMPLEMENT study [ACTRN012606000098538], with the aim of facilitating early peer-review of proposed methods and to provide a transparent statement of planned analyses.

## Methods

The IMPLEMENT study is a CRCT, with the clusters being a sample of 92 general practices of one or more GPs drawn from a sampling frame of 1,000 general practices within the state of Victoria, Australia. Participating practices will be randomised to either a control group or an intervention group. Enrolling an average of 25 patients per practice will yield 2,300 patients for inclusion in intention-to-treat analyses. A detailed description of the design of the CRCT and of proposed methods for sample selection, randomisation, and analysis of clinical outcomes are provided elsewhere [2].

Cost-effectiveness and cost-utility analyses will be conducted alongside the CRCT to quantify the additional costs (savings) and health gains associated with the implementation strategy as compared with dissemination alone from a societal perspective. Specific secondary aims will be to determine whether the incremental costs of the implementation strategy are outweighed by incremental cost-savings associated with any change in practice (*i.e*., whether implementing CPG for acute LBP is cost-saving as compared with dissemination alone), and to determine whether the implementation strategy dominates dissemination alone (*i.e*., less costly but of at least equivalent effect). The time horizon for inclusion of relevant costs and consequences is set at three months, consistent with the final scheduled follow-up of patients from the CRCT. That is to say, the economic evaluation is explicitly 'within-trial' and any subsequent extrapolation beyond the trial period will be treated as a separate research task.

The proposed economic evaluation will take a societal perspective in identifying, measuring and valuing all costs and consequences associated with development of the implementation strategy, delivery of the implementation strategy, and any subsequent changes in practice and subsequent health effects. Note, however, that the development and dissemination of the CPG for acute LBP have already taken place in Australia with the result that their associated costs and effects are common and invariant to both intervention and control groups. The incremental costs and effects associated with implementation are therefore likely to be of greatest interest to policy-makers and the proposed study will not explicitly calculate costs associated with development and dissemination of the CPG.

### Description of the comparator intervention

In 2003, the National Health and Medical Research Council (NHMRC) of Australia endorsed a CPG for acute LBP [1]. There is a strategy in place to disseminate the CPG for acute LBP. This strategy comprises development of user-friendly material for the target audiences (clinicians and consumers), a range of methods to access the information, publicising the availability of the materials, endorsement by professional and lay associations, and approval by the NHMRC. All documents are available electronically via the NHMRC website [1]. In addition, the summary (user-friendly) version of the review for clinicians, which includes the consumer information sheets, was distributed by mail to approximately 40,000 clinicians across Australia. While the comparator intervention closely approximates this 'existing practice' strategy, a printed copy of the guideline and a written reminder of how to access the electronic version of the CPG will be sent to control group practices after randomisation.

### Description of the evaluated intervention

In addition to access via the existing dissemination strategy, the intervention group will receive active implementation of the CPG for acute LBP. The GPs randomised to the intervention arm will receive an implementation strategy specifically designed to address the barriers and enablers for implementation of the CPG. The intervention will concentrate on delivering the CPG's key messages, namely that diagnostic x-rays are rarely necessary in the management of acute LBP and that remaining active reduces pain and disability. The intervention will consist of a series of facilitated face-to-face small group workshops. These workshops will involve a combination of didactic lectures and small group interaction. The delivery of the intervention will be co-ordinated by a project officer and delivered by members of the research team and external facilitators.

### Identification of health outcomes

The program logic of the evaluated intervention and the symptomatology of acute LBP suggest that specific dimensions of HRQoL, such as pain-related disability, physical function, and physical pain are most relevant in capturing variation in health outcomes. However, it is possible that a differential effect might arise between the evaluated intervention and the comparator with respect to dimensions of HRQoL other than physical disability or physical pain. The outcome measures specified below will therefore provide broad coverage of HRQoL.

### Measurement of health outcomes

The measures chosen to assess patient outcome are commonly used in trials of interventions for acute LBP and provide broad coverage of HRQoL, including those dimensions of HRQoL that are most likely to be relevant in identifying an effect attributable to the intervention.

### The Roland-Morris Disability Questionnaire (RDQ)

The RDQ is among the most widely used and well-validated measures of LBP-specific disability. The RDQ has high validity and reliability for use over the telephone and is recommended for inclusion in core sets of measures used in LBP [17]. The RDQ measures 24 activity limitations due to back pain. The RDQ score is calculated by adding up the number of items with positive responses, the scores therefore ranging from 0 (no disability) to 24 (maximum disability).

### Usual pain

An 11-point scale (0 = no pain to 10 = worst pain ever) has acceptable reliability and validity for self-reported assessment of pain [18].

### Assessment of Quality of Life (AQoL)

The AQoL can be used as either a psychometric instrument, yielding a descriptive measure of HRQoL, or a multi-attribute utility instrument that yields a preference-based measure of HRQoL. The AQoL descriptive system includes five latent dimensions with each dimension reflected in three items: illness (prescribed medicines, medication and aids, and medical treatment), independent living (self-care, household tasks, and mobility), social relationships (relationships with others, social isolation, and family role), physical senses (seeing, hearing, and communication) and psychological well-being (sleep, anxiety, and depression). Four of the five dimensions and 12 of the 15 items contribute to the AQoL's preference-based measure of HRQoL, with the illness dimension excluded because it was deemed indicative of an underlying health condition rather than the impact of that health condition on HRQoL [19]. The AQoL preference-based measure of HRQoL ranges from -0.04 to 1.00, where unity designates full health, zero designates death, negative scores designate states worse than death, and the lower bound of -0.04 designates the AQoL's 'all worst health state'. The validity and reliability of the AQoL Version 1.0 for the measurement of preference-based HRQoL has been demonstrated in the Australian general population [19,20]. The effect of mode of administration (mail versus telephone administration) on mean AQoL scores was neither clinically nor statistically significant [19].

Follow-up of patient level outcomes is scheduled for seven days and three months after their initial GP consultation for acute non-specific LBP. Table 1 provides a schedule of patient-level measures for the cost-effectiveness and cost-utility analyses. Due to the method chosen to recruit patients to the trial, it is not possible to obtain baseline observations for patient-level outcomes. Intervention effects for the cost-effectiveness analyses will be taken from the main analysis of RDQ and usual pain measures at day seven and three month follow-up, controlling for a pre-specified set of potential confounders [2].

**Table 1:** Schedule of measures for economic evaluation

**Outcome**	**Data collection**	**Timing**	**Source**	**Level**
RDQ	Telephone interview	7 days and 3 months after consultation	Patient	Patient
Usual pain	Telephone interview	7 days and 3 months after consultation	Patient	Patient
AQoL	Telephone interview	7 days and 3 months after consultation	Patient	Patient
X-ray referral	Data abstraction	3 months	GP case notes	Patient
Any imaging referral	Data abstraction	3 months	GP case notes	Patient
Health Service Utilisation	Telephone interview	7 days and 3 months after consultation	Patient	Patient
Direct costs of developing intervention	Data abstraction Interview	On completion of development	Admin records Project officers	Intervention
Direct costs of delivering intervention	Data abstraction Interview	On completion of delivery to all GPs	Admin records Project officers	Intervention

### Valuation of health outcomes

While the patient level outcomes described above are expected to capture all relevant dimensions of health outcome, there are a number of advantages in expressing the results of cost-effectiveness analyses in cost per quality adjusted life year (QALY) terms. Between-group differences in preference-based HRQoL weights derived from the AQoL will be combined with the period of time over which such differences persist to calculate effectiveness in QALY terms. Intervention effects with respect to AQoL scores will be estimated using methods specified for the main analysis of patient level clinical outcome measures, controlling for the same set of pre-specified set of potential confounders [2]. In the absence of detailed information as to the form of the time-trend in HRQoL for the target condition, patients in both groups will be assumed to track a linear path from their AQoL score at seven days to their AQoL score at three months and the incremental QALY gain calculated as the difference between curves for treatment and control groups. For the within-trial analysis, groups will be assumed equivalent prior to the seven day follow-up and post the three month follow-up.

### Identification of resource use

Incremental costs will reflect all resource use associated with development of the implementation strategy, delivery of the implementation strategy, and any subsequent changes in practice and subsequent health effects. All research and evaluation costs will be excluded from the cost analysis. Two questions arise in specifying the minimum dataset for the cost analysis. First, can any cost items be excluded from the base-case analysis without biasing our estimate of incremental costs? And, second, for which included costs should we directly collect data from either GPs or patients? Drummond *et al*. note that some costs may simply confirm a result that would be obtained by consideration of a narrower range of costs [13]. Volunteer caregiver time, patients' waiting time, and travel time to attend x-ray appointments and follow-up GP consults may fall into this category. Costs associated with development and dissemination of the CPG under existing practice are assumed to arise in equal magnitude for intervention and control groups, and are excluded from further consideration. Note, however, that the cost of sending the guideline and written reminders to control group practices is specific to the control group and will be included in the cost analysis. The scope of the within-trial cost analysis proposed here is also constrained by the design of the IMPLEMENT CRCT, with observation on relevant costs and consequences limited to three months from each patient's initial GP consultation.

Cost items associated with development of the implementation strategy include the cost of recruiting informants to assist development of the intervention, the cost of time in focus groups for informants and facilitators, the opportunity cost of interview and meeting rooms, the cost of time and equipment for analysis and interpretation of focus group data, and the cost of consultation with the GP advisory committee. Drummond *et al*. note the importance of identifying capital outlays that should be amortised over the lifetime of the asset [13]. Certainly the development of the implementation strategy amounts to a one-time investment in intellectual property that may find a repeated or broader use. Given repeated use, it would be inappropriate to apportion the entire cost of development to a single use. It would also be inappropriate to disregard the investment in intellectual property and the full cost must be estimated before it can be amortised. Here, the implementation strategy is likely to be specific to the LBP CPG, specific to the institutional context of Australia, specific to general practice, likely to have limited value in repeated use for the current cohort of practitioners, and may have an expiration date contingent upon the current technology or the existing evidence base. The cost of developing the implementation strategy will therefore be amortised under the assumption that the strategy will eventually be delivered to the entire cohort of Australian GPs with current Medicare provider numbers, but will bear no repeated or wider use.

Cost items associated with delivery of the implementation strategy include administrative costs associated with coordinating small group workshops, production of materials for workshops, the opportunity cost of venue use, the opportunity cost of travel time, and attendance at workshops for GPs, opportunity cost of pre- and post-workshop reflection for GPs, and labour costs associated with preparation/delivery/facilitation of workshops.

Cost items associated with change in clinical practice include direct and indirect health care costs, such as use of x-rays, over-the-counter or prescription analgesics, allied health or GP consults, and the time of volunteer or paid caregivers. Practice change is also expected to impact on direct and indirect costs outside the health sector, including waiting time and travel time to attend treatment, productivity gains due to a change in specific disability, and time lost from work associated with treatment.

### Measurement of resource use

In measuring resource use associated with development of the implementation strategy, delivery of the implementation strategy, and any subsequent changes in practice and subsequent health effects, data will be collected from the research team, from the enrolled practitioners and from the enrolled patients. Scheduled observations on resource use are listed in Table 1.

Resource use associated with the development of the implementation strategy will be costed based on financial and administrative records, as well as a detailed description of the development process obtained from the project manager and project officers. This will require recall over a relatively short period of time as to proportion of an equivalent full-time salary that project staff spent in development of the implementation strategy (as opposed to activities associated with research and evaluation such as administration/design of the CRCT). Administrative and financial records will provide data as to the number of informants, total person hours spent in focus groups and interviews for informants and facilitators, use of interview and meeting rooms, total person time for analysis/interpretation of findings, and total person hours for advisory committee members.

Resource use associated with the delivery of the implementation strategy will be estimated from administrative and financial records detailing resource use associated with the production of materials, total person hours spent in organising and facilitating workshops, total hours GP attendance, venue location and total hours venue hire, and practice location for attending GPs. Time spent by GPs in pre- and post-workshop reflection or self-education will be estimated based on a description of the intervention (rather than self-report) and varied in sensitivity analysis.

Resource use associated with a change in clinical practice and subsequent health effects will be based on patient self-report. While GPs may be in a position to report the percentage of LBP patients referred for x-ray, patients' use of allied health care and their use of over-the-counter analgesics cannot be obtained from enrolled practitioners. It is well-known that patient self-report becomes increasingly unreliable as the period of recall increases. "For example, one study found that 10% of patients failed to report that they had been hospitalized when they were interviewed approximately five months after discharge. Even if subjects remember that they have seen a physician or have gone to a hospital, they often will not know what services they received [21]." For longer periods of recall, memory aids such as patient diaries have proven useful in improving the reliability of self-report data. In the present study, the period of recall is just the period since baseline at each follow-up (seven days and three months). The use of patient diaries is not possible for the seven days prior to the post-consultation follow-up. Moreover, there is good reason to believe that recall with respect to use of allied health consults and over-the-counter medication is likely to be relatively accurate because these items are often paid as out-of-pocket costs rather than bulk-billed to Medicare or reimbursed directly from private insurance. It therefore seems sufficient to present a short questionnaire (see Table 2) to patients at each follow-up with the breadth and form of questions based on health-related actions items from the ABS National Health Survey (ABS 4801.0, 1995). This short questionnaire will also ask patients to nominate a category describing their usual main activity and to estimate the amount of time they spent away from their usual main activity due to illness or to attend treatment; providing the raw data for estimating productivity gains/losses and travel/waiting time (see Table 2). Self-report for professional and volunteer home care has not been requested in the questionnaire due to reliability concerns. Total hours of caregiver time will be conservatively estimated based on measures of LBP-related disability.

**Table 2:** Patient self-report of health service utilisation

INSTRUCTIONS: Thinking about your use of health services over the last (7 days/3 months)...
Have you received an x-ray in the last (7 days/3 months)? *(YES/NO/NOT SURE)*
*If yes*, how many times have you attended radiology for an x-ray in the last (7 days/last 3 months)?
Have you been hospitalised in the last (7 days/3 months)? *(YES/NO/NOT SURE)*
*If yes*, how many times have you been hospitalised in the last (7 days/last 3 months)?
Have you visited casualty/emergency in the last (7 days/3 months)? *(YES/NO/NOT SURE)*
*If yes*, how many times have you visited casualty/emergency in the last (7 days/last 3 months)?
Have you visited an outpatient or day clinic in the last (7 days/3 months)? *(YES/NO/NOT SURE)*
*If yes*, how many times have you visited an outpatient or day clinic in the last (7 days/3 months)?
Have you visited any GP in the last (7 days/3 months)? *(YES/NO/NOT SURE)*
*If yes*, how many times have you visited a GP in the last (7 days/3 months)?
Have you visited a medical/surgical specialist in the last (7 days/3 months)? *(YES/NO/NOT SURE)*
*If yes*, how many times have you visited a specialist in the last (7 days/3 months)?
Have you visited any physiotherapist in the last (7 days/3 months)? *(YES/NO/NOT SURE)*
*If yes*, how many times have you consulted a physiotherapist in the last (7 days/3 months)?
Have you visited any osteopath in the last (7 days/3 months)? *(YES/NO/NOT SURE)*
*If yes*, how many times have you visited an osteopath in the last (7 days/3 months)?
Have you visited any chiropractor in the last (7 days/3 months)? *(YES/NO/NOT SURE)*
*If yes*, how many times have you visited a chiropractor in the last (7 days/3 months)?
Have you visited any other health provider (OHP1) in the last (7 days/3 months)? *(YES/NO/NOT SURE)*
*If yes*, how many times have you visited OHP1 in the last (7 days/3 months)?
Have you visited any other health provider (OHP2) in the last (7 days/3 months)? *(YES/NO/NOT SURE)*
*If yes*, how many times have you visited OHP2 in the last (7 days/3 months)?
Have you used any prescription or over-the-counter medications in the last (7 days/3 months)? *(YES/NO/NOT SURE)*
*If yes*, how many different medications have you used in the last (7 days/3 months)?
Have you used any prescription or over-the-counter pain relievers in the last (7 days/3 months)?*(YES/NO/NOT SURE)*
*If yes*, on how many days have you taken pain relievers in the last (7 days/3 months)?
Have you used any prescription or over-the-counter sleeping medications in the last (7 days/3 months)? *(YES/NO/NOT SURE)*
*If yes*, on how many days have you taken sleeping medications in the last (7 days/3 months)?
INSTRUCTIONS: Thinking about your usual main activity over the last (7 days/3 months)...
What is your usual main activity?
*Full-time student*
*Part-time student*
*Employed*
*Unemployed*
*Not applicable*
How many hours would you usually spend on your main activity in a week?
*0 hours*
*1–15 hours*
*16–24 hours*
*25–34 hours*
*40 hours*
*41–48 hours*
*49 hrs or more*
Have you spent time away from your usual main activity due to illness or to attend treatment in the last (7 days/3 months)?*(YES/NO/NOT SURE)*
*If yes*, how many full days away from usual main activity due to illness in the last (7 days/3 months)?
And how many hours away from usual main activity to attend treatment in the last (7 days/3 months)?

### Valuation of resource use

Unit costs for health service resource use will be as per the Manual of Resource Items for use in submissions to the Commonwealth of Australia's Pharmaceutical Benefits Advisory Committee [22]. Resource use of marketed goods and services outside the health sector and not included in the manual will be valued at market prices. Unmarketed goods and services such as travel time and the time of volunteer caregivers will be costed using opportunity cost prices. Productivity gains/losses will be valued as described below. Intervention effects with respect to total cost will be estimated using methods specified for the main analysis of patient level clinical outcome measures, controlling for the same set of pre-specified set of potential confounders [2].

### Productivity gains and losses

Production losses are most frequently valued via the human capital approach, whereby the actual gross earnings of those in paid employment are used to estimate the value of time absent from work due to treatment or illness [13]. Note, however, that a significant minority of enrolled patients are expected to be retirees or engaged in home duties as their major activity such that the human capital approach would underestimate the value of lost production. To avoid bias and certain unpalatable equity implications of the human capital approach, a proxy (*e.g*., average wage rates, cost of services such as childcare or cleaning services required to replace role of homemaker) is frequently used to value the unpaid work of retirees, homemakers, and the unemployed. For example, the Bureau of Transport Economics [23] estimated the value of lost production in household and community sectors using the ABS Time Use Survey [24] and average earnings by age group. The proposed study will adopt a similar approach, deriving the cost of a week out-of-role as the product of the average ordinary hourly wage rate taken from the ABS Labour Price Index for the year of study completion [25], and the average number of hours per week spent on unpaid work in household and community sectors by age group taken from the ABS Time Use Survey [24].

Due to the relatively short duration for an episode of acute non-specific LBP and the relatively short duration of follow-up, the difference between human capital and friction cost [13] estimates of productivity gains/losses in the present study is expected to be negligible. The friction cost approach would censor productivity losses that accrue beyond the minimum period of time required for the replacement of effective labour. While Hoeijenbos *et al*. employed a friction cost approach to the estimation of productivity gains/losses in their study of guideline implementation for non-specific LBP [9], productivity losses were not censored until 22 weeks of absence under the assumption that recruitment and training could not routinely be accomplished within a shorter time-frame. For this reason, the proposed study will not censor productivity losses that arise within the study period.

### Adjustment for differential timing

All costs will be inflated to current Australian dollars for the year of study completion. While costs and benefits associated with delivery of the implementation strategy and any subsequent within-trial changes in practice are expected to accrue within a 12 month period, costs associated with development of the implementation strategy are expected to accrue up to 24 months prior to costs associated with delivery of the implementation strategy and any subsequent within-trial changes in practice. All costs and benefits will be converted to present values using an annual discount rate of 5% in the base-case, and annual rates of 3% and 7% in sensitivity analysis.

### Incremental analysis

Results from the economic evaluation will be expressed as: additional costs (savings) per point difference in RDQ at seven days and three months; additional costs (savings) per point difference in usual pain at seven days and three months; and additional costs (savings) per QALY gained.

### Uncertainty

Cost-effectiveness acceptability curves (CEACs) will be used to quantify and visualise sampling and parameter uncertainty. The CEAC is derived from the joint density or joint distribution of incremental costs (ΔC) and incremental effects (ΔE) for the intervention of interest against a comparator, and represents the proportion of the density where the intervention is cost-effective for a range of willingness to pay thresholds (λ). The joint density will be obtained via non-parametric bootstrapping from the distribution of observed cost/effect pairs. Because the base-case analysis will include all cost items listed above, a separate CEAC will be calculated using best estimate and upper/lower bound estimates for travel time, time of volunteer caregivers, productivity gains and for other uncertain parameters such as the discount rate. Sensitivity analysis on the CEAC will also adjust estimates of incremental costs and incremental benefits for practice characteristics and patient characteristics to consider the external validity of study findings.

## Conclusion

Publication and peer-review of protocols for economic evaluation is advisable for much the same reasons as has been advocated elsewhere for trial protocols [26]. Publication bias and the 'mining' or 'gaming' of analyses are no less a problem for economic analyses than they are for experimental and observational studies of efficacy and effectiveness [14,15]. The protocol provided here registers our intent to conduct an economic evaluation alongside the IMPLEMENT study, allows peer-review of proposed methods and provides a transparent statement of planned analyses.

## Ethical review

Ethical approval for this trial was obtained from the Monash University Standing Committee on Ethics in Research involving Humans (2006/047).

## Competing interests

Sally Green is a member of the editorial board of Implementation Science. The remaining authors declare that they have no competing interests.

## Authors' contributions

DM participated in the design of the trial-based economic evaluation and drafted the protocol. SF, JM, DO'C and SG participated in the design of the trial-based economic evaluation and suggested edits and revisions to the protocol. All authors read and approved the final manuscript.
